# IgM-enriched immunoglobulins (Pentaglobin) may improve the microcirculation in sepsis: a pilot randomized trial

**DOI:** 10.1186/s13613-019-0609-5

**Published:** 2019-12-03

**Authors:** Roberta Domizi, Erica Adrario, Elisa Damiani, Claudia Scorcella, Andrea Carsetti, Paolo Giaccaglia, Erika Casarotta, Vincenzo Gabbanelli, Simona Pantanetti, Elena Lamura, Silvia Ciucani, Abele Donati

**Affiliations:** 1 Anesthesia and Intensive Care Unit, Department of Biomedical Sciences and Public Health, Università Politecnica delle Marche, via Tronto 10/a, 60126 Torrette di Ancona, Italy; 2 Hospital Pharmacy, Azienda Ospedaliera Universitaria “Ospedali Riuniti Umberto I-Lancisi-Salesi” of Ancona, via Conca 71, 60126 Torrette di Ancona, Italy

**Keywords:** Immunoglobulins, Pentaglobin, Sepsis, Microcirculation, Immunomodulation

## Abstract

**Background:**

Polyclonal or IgM-enriched immunoglobulins may be beneficial during sepsis as an adjuvant immunomodulatory therapy. We aimed to test whether the infusion of IgM-enriched immunoglobulins improves microvascular perfusion during sepsis.

**Methods:**

Single-centre, randomized, double-blind, placebo-controlled phase II trial including adult patients with a diagnosis of sepsis or septic shock for less than 24 h. Patients received an intravenous infusion of 250 mg/kg (5 mL/kg) per day of IgM-enriched immunoglobulins (Pentaglobin, *n* = 10) for 72 h or placebo (NaCl 0.9%, *n* = 9). At baseline and after 24 and 72 h of infusion, the sublingual microcirculation was assessed with Incident Dark Field videomicroscopy. Thenar near-infrared spectroscopy (NIRS) was applied with a vascular occlusion test to assess tissue oxygenation and microvascular reactivity. Levels of interleukin (IL) 1-beta, IL-6, IL-8, IL-10 and tumour necrosis factor alpha were measured in the serum.

**Results:**

The perfused vessel density (PVD) for small vessels (diameter < 20 micron) increased in the Pentaglobin group (from 21.7 ± 4.7 to 25.5 ± 5.1 mm/mm^2^) and decreased in the placebo group (from 25 ± 5.8 to 20.7 ± 4.1 mm/mm^2^, *p* for interaction < 0.001, two-way analysis of variance). The absolute between-group difference at 72 h was 4.77 (standard error 2.34), *p* = 0.140. The microvascular flow index for small vessels increased at 24 h in the Pentaglobin group (from 2.68 [2.38–2.78] to 2.93 [2.82–3], *p* < 0.01) and decreased at 72 h in the placebo group (from 2.83 [2.60–2.97] to 2.67 [2.48–2.73], *p* < 0.05). Changes in general parameters, cytokines and NIRS-derived parameters were similar between the two groups, except for IL-6 and IL-10 that significantly decreased at 72 h only in the Pentaglobin group.

**Conclusions:**

A 72-h infusion of IgM-enriched immunoglobulins (Pentaglobin) in patients with sepsis or septic shock may be associated with an increase in sublingual microvascular perfusion. Further studies are needed to confirm our findings.

*Trial registration* NCT02655133, www.ClinicalTrials.gov, date of registration 7th January 2016, https://www.clinicaltrials.gov/ct2/show/NCT02655133.

## Background

Sepsis is a major healthcare problem, with high mortality and morbidity: even if some reports showed a decline in crude hospital mortality in the last decade [1], sepsis survivors remain at higher risk of infections, cardiovascular events, acute renal failure or the development of new physical disability or cognitive impairment [2]. At present, sepsis treatment is non-specific and mainly based on antibiotics and hemodynamic support [3].

Sepsis is characterized by a dysregulated host response to an infection, with uncontrolled activation of both pro- and anti-inflammatory pathways [4]. Increasing evidence suggests that a state of immunoparalysis is mainly responsible for adverse outcome. A recent meta-analysis showed a significant reduction in circulating B cells and immunoglobulin M (IgM) levels in sepsis non-survivors as compared to survivors [5]. The administration of polyclonal or IgM-enriched immunoglobulins as an adjuvant immunomodulatory therapy gave encouraging results in both pre-clinical and clinical studies [6], although the evidence supporting a reduction in mortality is still too weak to justify a widespread use in septic patients [7]. The potential benefits of immunoglobulins (especially IgM-enriched preparations) are related not only to their anti-inflammatory activity (pathogen recognition and clearance, toxin scavenging, inhibition of inflammatory mediators production, cytokine neutralization, complement-scavenging properties) but also to their anti-apoptotic effects on immune cells [8]. Pre-clinical studies showed a potential role in the regulation of endothelial cell function, leukocyte adhesion and capillary perfusion [9, 10]. Nonetheless, no clinical studies exist that evaluated the microvascular effects of immunoglobulins in septic patients.

We hypothesized that the intravenous administration of IgM-enriched immunoglobulins in patients with sepsis as an adjunctive therapy could improve microvascular perfusion. This may result in better tissue oxygenation and preserved organ function. The primary goal of this study was to evaluate whether the infusion of IgM-enriched immunoglobulins was able to increase the sublingual perfused vessel density (PVD) after 72 h as compared to a placebo. Secondary endpoints were parameters of microcirculatory flow quality, peripheral (skeletal muscle) tissue oxygenation and microvascular reactivity.

## Methods

This single-centre, randomized, double-blind, placebo-controlled phase II trial was conducted in the Intensive Care Unit of Azienda Ospedaliera Universitaria “Ospedali Riuniti” of Ancona in Italy. The study protocol was approved by the local ethics committee (Comitato Etico Regionale Marche) and registered in www.ClinicalTrials.gov (Identifier: NCT02655133, date of registration 7th January 2016, https://www.clinicaltrials.gov/ct2/show/NCT02655133). Written informed consent was obtained before enrolment from all patients or their legal representatives in accordance with current Italian legislation. A deferred consent procedure was applied in case of temporary inability.

This manuscript adheres to the 2010 Consolidated Standards of Reporting Trials statement.

### Participants

We included adult (≥ 18 years old) patients with severe sepsis or septic shock according to the 2001 International Sepsis Definition Conference criteria [11], as the original study protocol was approved before the publication of the Sepsis-3 definitions [12]. Severe sepsis was defined by the presence of at least one sepsis-induced organ dysfunction; septic shock was defined as persistent hypotension despite adequate fluid resuscitation, requiring vasopressor infusion [11]. Nonetheless, the term “sepsis” (instead of severe sepsis) will be used hereafter, as the current concept of sepsis now includes the presence of an organ dysfunction induced by a dysregulated response to infection [12]. In addition, the term “septic shock” will refer to a condition of persistent arterial hypotension despite adequate fluid resuscitation and lactate levels > 2 mmol/L, based on the current definition [12]. All patients were enrolled within the first 24 h of sepsis development. Exclusion criteria were: contraindications to immunoglobulin treatment; sepsis/septic shock for more than 24 h; history of chronic renal failure; life expectancy < 24 h; lack of informed consent; pregnancy; factors impeding the sublingual microcirculation evaluation (recent oral surgery or maxillo-facial trauma); inclusion in other interventional studies. Patients with a history of chronic renal failure were excluded since previous studies showed a higher risk of osmotic-induced renal damage following intravenous immunoglobulins infusion in those with pre-existing renal insufficiency [13].

### Interventions

Patients were randomly assigned to one of two study groups. Patients in the Pentaglobin group received 250 mg/kg (5 mL/kg) per day of IgM-enriched immunoglobulins (Pentaglobin, Biotest Pharma GmbH, Dreieich, Germany) as a continuous intravenous infusion for 72 h. Patients in the placebo group received the same volume of normal saline solution (NaCl 0.9%) within a period of 72 h. Saline solution was chosen as the placebo treatment as being an inert substance with no expected biological effect at the volume infused in this study. A simple randomization was performed by a pharmacist through sealed envelopes with a 1:1 allocation ratio. The study treatment or placebo was then provided by the Hospital Pharmacy in identical bottles masked in opaque green plastic bags: neither the attending physicians nor the investigators nor the patient were aware of the study group. All other therapies (including fluids and vasoactive agents) were provided according to individual needs and based on the Surviving Sepsis Campaign guidelines [3].

### Measurements

All measurements were performed at baseline, 24 h after starting Pentaglobin/placebo infusion and at the end of infusion (72 h). Mean arterial pressure (MAP), heart rate (HR) and norepinephrine requirements were recorded. Arterial and central venous blood samples were collected in order to measure blood gases, arterial lactate, haemoglobin (Hb), white blood cell (WBC) count, procalcitonin, interleukin (IL) 1 beta, IL-6, IL-8, IL-10, tumour necrosis factor alpha (TNF-α). The Simplified Acute Physiology Score (SAPS) II, Acute Physiology and Chronic Health Evaluation (APACHE) II score were obtained at admission, and Sequential Organ Failure Assessment (SOFA) score at the study time-points.

The sublingual microcirculation was evaluated by means of incident dark field (IDF) videomicroscopy (Cytocam, Braedius Medical, Amsterdam, The Netherlands). The Cytocam-IDF is a third-generation handheld microscope that enables real-time in vivo visualization of the microcirculation. It consists of an illumination unit based on IDF imaging with a 4× magnification lens. The illumination light is emitted with a short pulse time of 2 ms (synchronized with a computer-controlled image sensor) and a wavelength of 548 nm, ensuring the highest absorption of oxyhaemoglobin and deoxyhaemoglobin, whereby flowing red blood cells are visible within the vessels as dark moving globules against a clear background [14]. After removal of secretions with a gauze, the probe was gently applied on the sublingual mucosa. At least 5 videos from different areas were recorded with adequate contrast and focus, and all efforts were made to avoid pressure artifacts. The image quality was checked offline [15], and videos of inadequate quality were discarded. Three videos per time-point were analysed offline with dedicated software (Automated Vascular Analysis 3.2, Microvision Medical, Amsterdam, NL) [16]. In brief, the image was divided by three equidistant horizontal and three equidistant vertical lines; the De Backer score was calculated as the number of vessels crossing the lines divided by the total length of the lines. For each vessel crossing the lines, perfusion was categorized as continuous, sluggish, intermittent or absent. The percentage of perfused vessels (PPV) was estimated as follows: 100 * [(total number of grid crossings − [no flow + intermittent flow])/total number of grid crossings] [16]. The total vessel density (TVD) was calculated as the total length of vessels divided by the total area of the image. The perfused vessel density (PVD) was estimated by multiplying TVD by PPV as estimated with the De Backer method. The microvascular flow index (MFI) was calculated semiquantitatively as described elsewhere [16]. The flow heterogeneity index (FHI) was also calculated as the highest MFI minus the lowest MFI, divided by the mean MFI, providing an index of heterogeneous microcirculatory perfusion [16]. The analysis was focused on small vessels (≤ 20 µ in diameter).

Near-infrared spectroscopy (NIRS) (InSpectra™ Model 650; Hutchinson Technology Inc., Hutchinson, MN, USA) with a 15-mm-sized probe was used to measure microvascular oxygen saturation (StO_2_) and tissue Hb index (THI) on the thenar eminence at baseline and during a vascular occlusion test (VOT) [17]. The StO_2_ downslope (%/min) was calculated as an index of oxygen consumption, while the StO_2_ upslope (%/min) and the area under the curve (AUC) of the hyperemic response were obtained to assess microvascular reactivity [17].

### Sample size calculation

The sample size was calculated based on the primary outcome of the study, i.e. we hypothesized that the infusion of Pentaglobin would be able to induce an increase in the PVD at 72 h. We calculated that the inclusion of 9 patients per group would be sufficient to detect a difference of at least 4 mm/mm^2^ (standard deviation: 3 mm/mm^2^) [18] between the two groups at 72 h with a power of 80% and an alpha error of 0.05. Ten patients per group were enrolled in total to allow for dropouts.

### Statistical analysis

This was performed using GraphPad Prism version 6 (GraphPad Software, La Jolla, CA, USA) and IBM Statistical Package for Social Science version 21 (Armonk, NY: IBM Corp.). Normality of distribution was checked using the Kolmogorov–Smirnov test. The data were expressed as mean ± standard deviation (SD) for normally distributed variables or median [1st–3rd quartiles] for non-normally distributed variables. For normally distributed variables, we applied a two-way analysis of variance (ANOVA) for repeated measures to test the effect of treatment (Pentaglobin versus Placebo) and time on the variables of interest. A Sidack’s post hoc test was used for multiple comparisons. For non-normally distributed variables, the Mann–Whitney *U* test was applied to evaluate differences between the two groups at each time-point and the Friedman test with the Dunn’s test for multiple comparisons was applied to evaluate differences between time-points in each group. Since we found significant inter-individual variability in microcirculatory parameters at baseline, we performed a secondary analysis by calculating the delta values (changes from baseline at 24 and 72 h) in each group and performed an analysis of covariance (ANCOVA) for repeated measures to evaluate the interaction between the factors “time” and “treatment” by controlling for the baseline value of the outcome of interest (in order to correct for the “regression to the mean” phenomenon), with the Bonferroni post hoc test to assess differences between the two group at each time-point. The Pearson *r* (or the Spearman rho) was calculated to evaluate correlation between variables. A two-tailed *p* < 0.05 was used to define statistical significance.

## Results

**Fig. 1:**
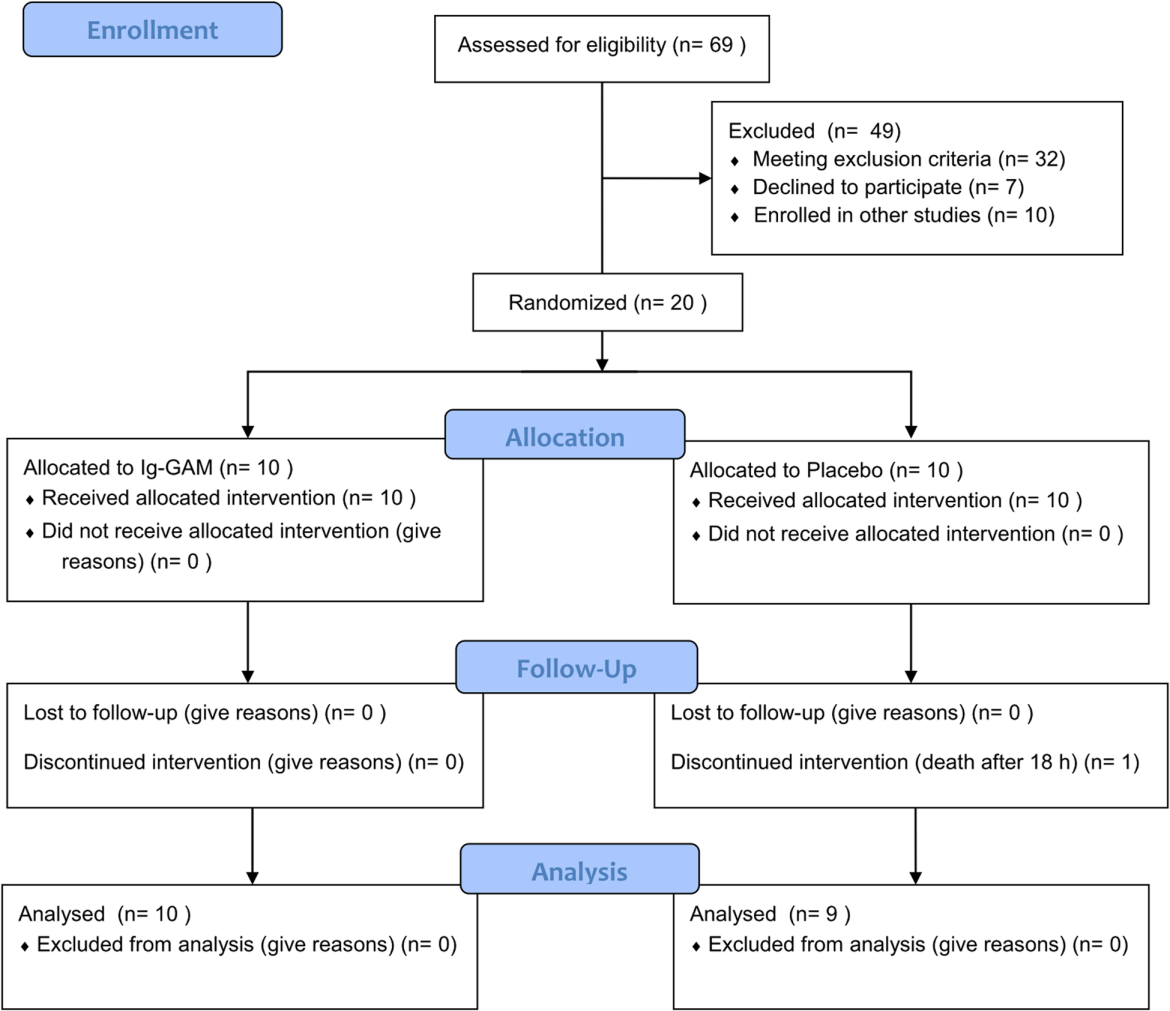
Study flow chart

From January 2016 to December 2017, 20 patients were enrolled in the study and randomized to receive Pentaglobin or placebo. One patient in the placebo group died 18 h after randomization, leaving 19 patients in total for the final analysis (Fig. 1).

Baseline characteristics for the two study groups are reported in Table 1.

**Table 1:** Baseline characteristics

Patient demographics	Pentaglobin (*n* = 10)	Placebo (*n* = 9)	*p*
Age (years)	62 ± 20	67 ± 16	0.545
Gender (nr of males, %)	7 (70%)	8 (89%)	0.582
Comorbidities (*n*, %)			0.659
Arterial hypertension	4 (40%)	3 (33%)	
Cardiopathy	1 (10%)	1 (11%)	
Diabetes	2 (20%)	1 (11%)	
Malignancy	3 (30%)	2 (22%)	
SAPS II (admission)	42 ± 17	45 ± 12	0.690
APACHE II (admission)	17 ± 8	21 ± 7	0.243
SOFA (admission)	9 ± 4	10 ± 3	0.663
Source of sepsis (*n*, %)			0.532
Abdominal	4 (40%)	2 (22%)	
Pulmonary	3 (30%)	2 (22%)	
Uro-genital	1 (10%)	3 (33%)	
Soft tissues	1 (10%)	2 (22%)	
Other	1 (10%)	0 (0%)	
Multi-drug-resistant pathogen (*n*, %)	4 (40%)	5 (56%)	0.656
Shock^a^ (*n*, %)	3 (30%)	2 (22%)	0.999

*SAPS* Simplified Acute Physiology Score, *APACHE* Acute Physiology and Chronic Health Evaluation, *SOFA* Sequential Organ Failure Assessment

^a^Persistent arterial hypotension despite adequate fluid resuscitation and hyperlactatemia (lactate levels > 2 mmol/L)

**Fig. 2:**
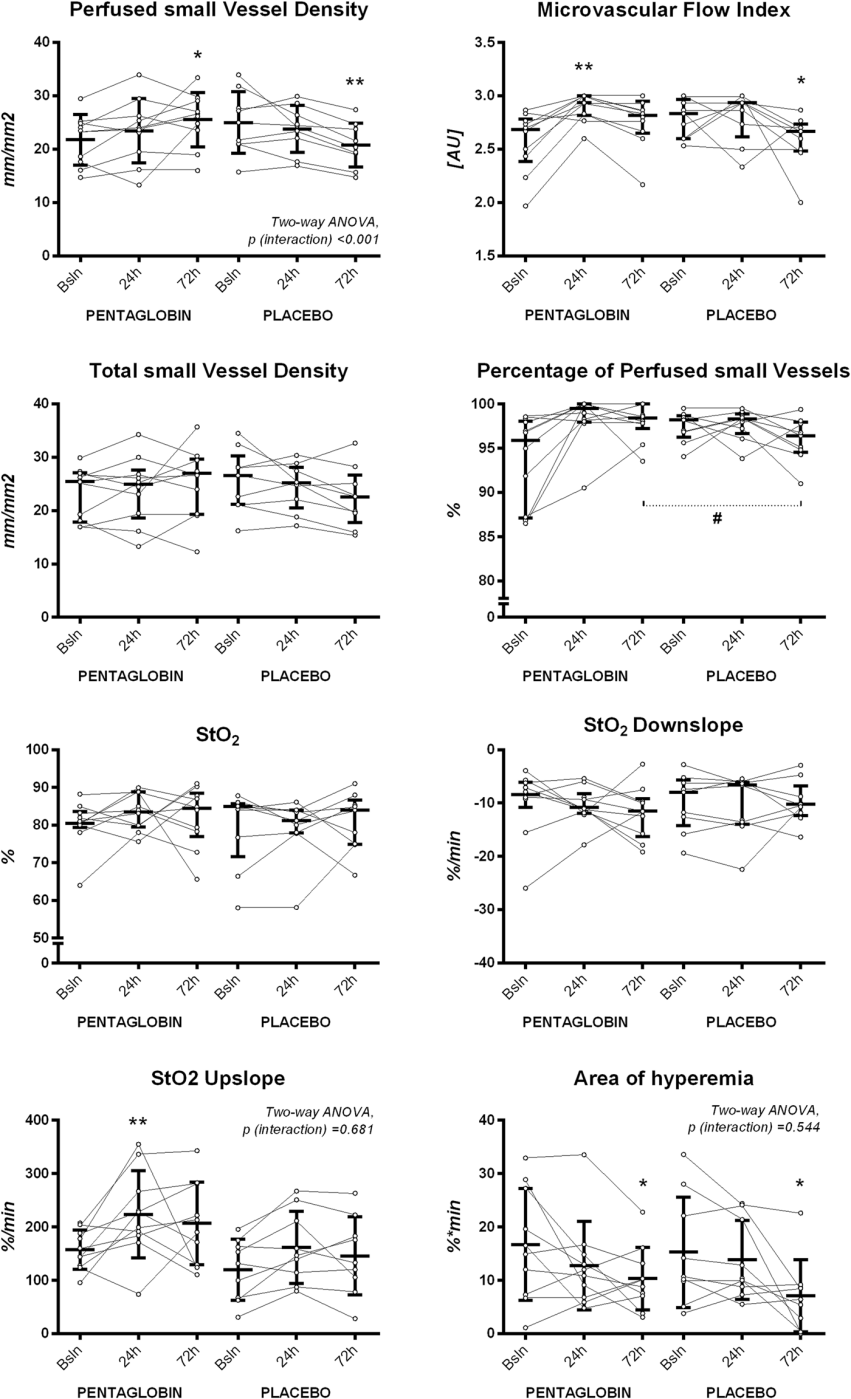
Comparison of microcirculatory and NIRS-derived parameters. Data are expressed as mean and standard deviation of median [interquartile range]; lines indicate individual changes. *p < 0.05, **p < 0.01 versus baseline, Two-way ANOVA for repeated measures with Sidack’s post hoc test or Friedman test with Bonferroni post hoc test, as appropriate. ^#^*p* < 0.05 versus placebo, Two-way ANOVA for repeated measures with Sidack’s post hoc test or Mann–Whitney *U* test, as appropriate

Changes in sublingual microvascular and NIRS-derived parameters are shown in Fig. 2 and Table 2. A two-way ANOVA showed a significant interaction effect of treatment and time on the PVD with an *F* ratio of *F* (degree of freedom = 2, error = 34) = 9.84 (*p* < 0.001). The Sidack’s post hoc test showed that the PVD was increased at 72 h in the Pentaglobin group (*p* < 0.05 versus baseline), while it was reduced in the Placebo group (*p* < 0.01 versus baseline). The between-group comparison at 72 h showed an absolute difference of 4.77 (standard error = 2.34), *p* = 0.140 (Student’s *t* test, *p* = 0.039). A comparison of delta values (adjusted for the baseline value) showed opposite variations of the PVD at 72 h (Pentaglobin: +3.8 ± 3.8, versus Placebo: − 4.2 ± 4.7, *p* = 0.003, Additional file 1, Fig. 3). The MFI was increased at 24 h in the Pentaglobin group, while it decreased at 72 h in the placebo group (Fig. 2, Table 2) and the comparison of delta values showed divergent changes at 72 h (+ 0.2 ± 0.2 versus − 0.2 ± 0.2, *p* = 0.035, Additional file 1). The PPV was higher in the Pentaglobin group as compared to the placebo group at 72 h (Fig. 2); however, variations from baseline did not differ between the two groups (Additional file 1). An example of sublingual microcirculation before and 72 h after Pentaglobin infusion is shown in Fig. 4.

**Table 2:** Changes in sublingual microcirculation and NIRS-derived parameters

	Baseline	24 h	72 h	*p* (time)^a^	*p* (interaction)^b^
PVDs (mm/mm^2^)				0.869	< 0.001
Pentaglobin (*n* = 10)	21.7 ± 4.7	23.4 ± 6.0	25.5 ± 5.1*		
Placebo (*n* = 9)	25.0 ± 5.8	23.8 ± 4.4	20.7 ± 4.1**		
MFIs (AU)					–
Pentaglobin (*n* = 10)	2.68 [2.38–2.78]	2.93 [2.82–3.00]**	2.82 [2.65–2.95]	0.002	
Placebo (*n* = 9)	2.83 [2.60–2.97]	2.93 [2.62–2.93]	2.67 [2.48–2.73]*	0.016	
TVDs (mm/mm^2^)					–
Pentaglobin (*n* = 10)	25.5 [17.9–27.1]	24.9 [18.7–27.6]	27.0 [19.3–29.6]	0.436	
Placebo (*n* = 9)	26.6 [21.1–30.3]	25.2 [20.5–28.1]	22.5 [17.8–26.6]	0.154	
De Backer score (n/mm)				0.887	0.144
Pentaglobin (*n* = 10)	13.1 ± 2.2	13.3 ± 3.4	14.2 ± 2.7		
Placebo (*n* = 9)	13.9 ± 2.8	13.2 ± 2.4	12.8 ± 2.7		
PPVs (%)					–
Pentaglobin (*n* = 10)	96 [87–98]	99 [98–100]	98 [97–100]	0.050	
Placebo (*n* = 9)	98 [96–99]	98 [97–99]	96 [94–98]^#^	0.154	
FHI (AU)					–
Pentaglobin (*n* = 10)	0.31 [0.16–0.45]	0.03 [0–0.13]	0.07 [0–0.18]	0.032	
Placebo (*n* = 9)	0.18 [0.03–0.30]	0.07 [0.07–0.18]	0.11 [0.03–0.26]	0.495	
StO_2_ (%)					–
Pentaglobin (*n* = 10)	80 [79–84]	84 [79–89]	84 [77–88]	0.316	
Placebo (*n* = 9)	85 [72–86]	81 [78–84]	84 [75–87]	0.658	
StO_2_ downslope (%/min)					–
Pentaglobin (*n* = 10)	− 8.4 [− 10.8, − 6.1]	− 10.9 [− 11.9, − 8.2]	− 11.5 [− 16.3, − 9.1]	0.367	
Placebo (*n* = 9)	− 8.0 [− 14.2, − 5.7]	− 6.6 [− 13.9, − 6.1]	− 10.2 [− 12.3, − 6.7]	0.813	
StO_2_ upslope (%/min)				0.005	0.681
Pentaglobin (*n* = 10)	157 ± 37	224 ± 82**	207 ± 77		
Placebo (*n* = 9)	120 ± 57	162 ± 68	146 ± 73		
Area of hyperemia (%*min)				0.002	0.544
Pentaglobin (*n* = 10)	16.7 ± 10.5	12.7 ± 8.3	10.3 ± 5.9*		
Placebo (*n* = 9)	15.3 ± 10.3	13.9 ± 7.4	7.1 ± 6.8*		
THI (AU)					–
Pentaglobin (*n* = 10)	12 [9–15]	11 [8–12]	10 [8–14]	0.601	
Placebo (*n* = 9)	11 [7–13]	10 [7–13]	8 [7–12]	0.654	

Data are expressed as mean ± standard deviation or median [1st–3rd quartile], as appropriate

*NIRS* near-infrared spectroscopy, *PVD* perfused vessel density, *MFI* microvascular flow index, *TVD* total vessel density, *PPV* percentage of perfused vessels, *FHI* flow heterogeneity index, *StO*_*2*_ tissue oxygen saturation, *THI* tissue haemoglobin index, *AU* arbitrary units

^a^Two-way analysis of variance for repeated measures (testing the effect of time) or Friedman test, as appropriate

^b^Two-way analysis of variance for repeated measures (testing for the interaction between time and treatment), when applicable

* *p* < 0.05, **p < 0.01 versus baseline, Two-way analysis of variance for repeated measures with Sidack’s post hoc test or Friedman test with Dunn’s post hoc test for multiple comparisons, as appropriate

^#^*p* < 0.05 versus Pentaglobin group, Mann–Whitney *U* test

**Fig. 3:**
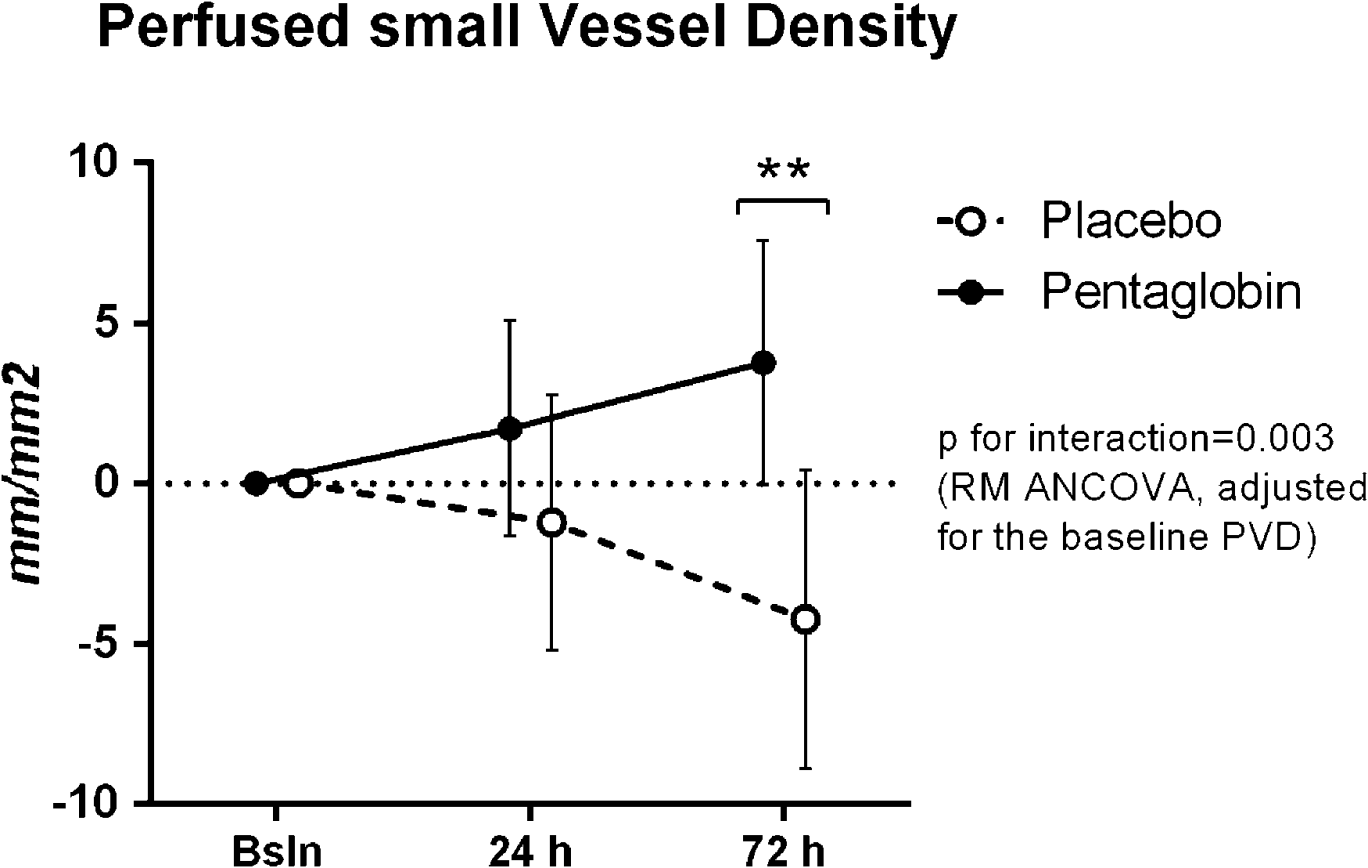
Comparison of the delta values (variations from baseline) for the perfused vessel density

**Fig. 4:**
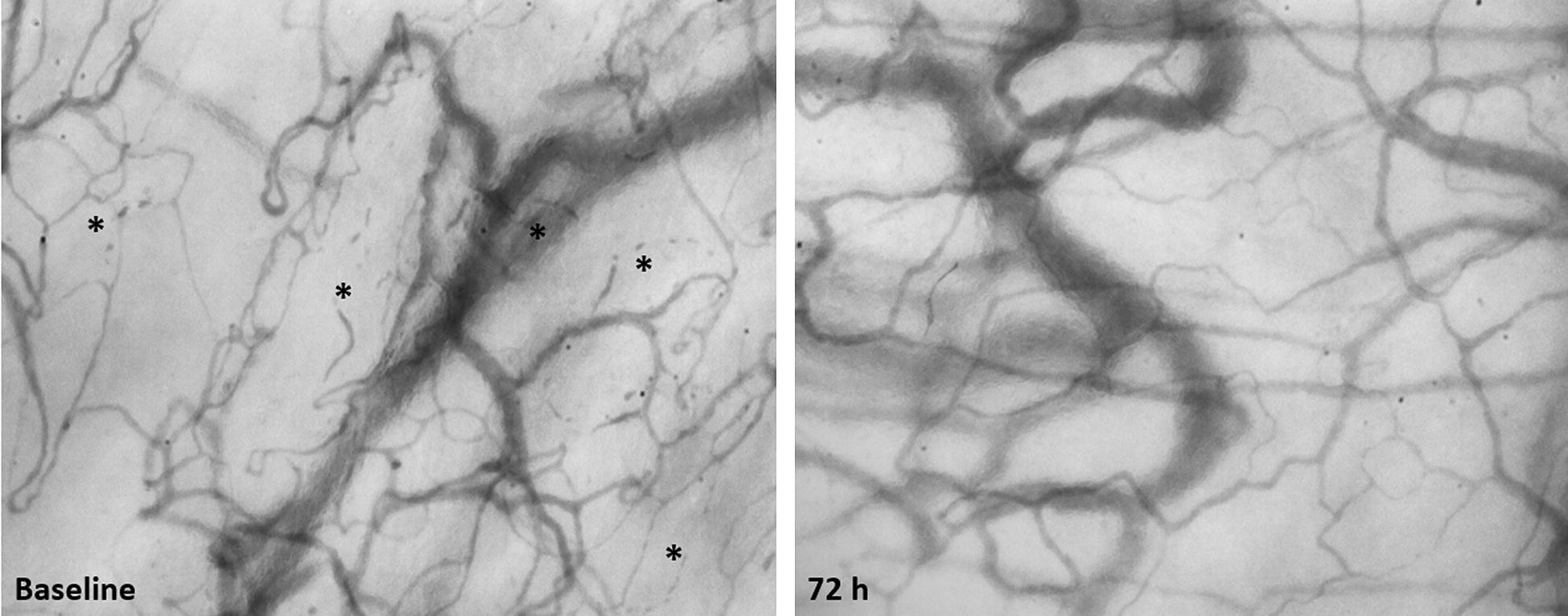
Images of the sublingual microcirculation of a patients at baseline and after 72 h of Pentaglobin infusion. Non-perfused vessels are indicated with stars

Two-way ANOVA showed significant effects of both time (with an *F* (2, 34) 6.29, *p* = 0.005) and treatment (with an *F* (1, 17) 4.51, *p* = 0.049) on the StO_2_ upslope, with no significant interaction (*p* = 0.681). Changes from baseline were similar between the two groups (Additional file 1). We found a significant effect of time on the area of hyperemia with an *F* (2, 34) 7.19 (*p* = 0.002), but variations over time did not differ between the two groups (Fig. 2, Additional file 1). No other differences were observed for microvascular and NIRS-derived parameters.

The administration of Pentaglobin did not induce any significant variation in MAP or HR, while norepinephrine dosage was decreased in the placebo group at 72 h (Table 3). A greater decrease in central venous O_2_ saturation (ScvO_2_) was found in the placebo group compared with the Pentaglobin group at 24 h (Table 3). Changes in the other parameters and SOFA score were similar between the two groups. Similarly, changes in WBC count, procalcitonin and cytokine levels in the Pentaglobin group did not differ from those in the placebo group, although a significant decrease in IL-6 and IL-10 at 72 h was only found in the Pentaglobin group (Table 4). ICU-mortality was similar between the two groups (20% in the Pentaglobin group and 22% in the placebo group, *p* = 0.999), as well as the ICU length of stay (19 ± 13 days in the Pentaglobin group versus 16 ± 12 days in the placebo group, *p* = 0.649).

**Table 3:** Changes in hemodynamic, blood gas parameters and organ function

	Baseline	24 h	72 h	*p* (time)^a^	*p* (interaction)^b^
MAP (mmHg)				0.083	0.309
Pentaglobin (*n* = 10)	80 ± 14	82 ± 9	86 ± 15		
Placebo (*n* = 9)	85 ± 15	75 ± 17	89 ± 13		
HR (bpm)				0.662	0.169
Pentaglobin (*n* = 10)	84 ± 23	85 ± 11	90 ± 21		
Placebo (*n* = 9)	95 ± 19	89 ± 30	80 ± 25		
Norepinephrine tartrate (*n*, mcg/kg/min)					–
Pentaglobin (*n* = 10)	8, 0.13 [0.03–0.39]	8, 0.12 [0.03–0.23]	7, 0.11 [0–0.38]	0.616	
Placebo (*n* = 9)	8, 0.38 [0.23–0.70]	8, 0.50 [0.14–0.70]	6, 0.10 [0–0.40]*	0.004	
Arterial pH				0.110	0.377
Pentaglobin (*n* = 10)	7.43 ± 0.07	7.42 ± 0.06	7.45 ± 0.07		
Placebo (*n* = 9)	7.45 ± 0.03	7.47 ± 0.05	7.49 ± 0.07		
PaO_2_/FiO_2_ (mmHg)				0.875	0.474
Pentaglobin (*n* = 10)	300 ± 89	311 ± 151	320 ± 89		
Placebo (*n* = 9)	311 ± 89	280 ± 74	262 ± 85		
ScvO_2_ (%)				< 0.001	0.053
Pentaglobin (*n* = 10)	80 ± 5	78 ± 10	72 ± 9*		
Placebo (*n* = 9)	79 ± 9	68 ± 9**^#^	71 ± 7*		
Base excess (mEq/L)				< 0.001	0.659
Pentaglobin (*n* = 10)	0.4 ± 4.9	2.5 ± 3.8	5.6 ± 4.9**		
Placebo (*n* = 9)	1.2 ± 4.7	4.0 ± 4.8	5.1 ± 4.4*		
Arterial lactate (mmol/L)					–
Pentaglobin (*n* = 10)	1.7 [1.3–3.5]	1.5 [1.0–2.2]	1.9 [0.9–2.7]	0.682	
Placebo (*n* = 9)	1.6 [1.0–2.2]	1.4 [1.0–2.2]	1.2 [1.1–1.7]	0.755	
Haemoglobin (g/dL)				0.460	0.356
Pentaglobin (*n* = 10)	10.4 ± 1.5	10.5 ± 0.9	9.6 ± 0.9		
Placebo (*n* = 9)	9.8 ± 1.8	10.1 ± 1.3	10.0 ± 1.1		
Platelets (*10^3^/mmc)				0.170	0.036
Pentaglobin (*n* = 10)	158 ± 98	141 ± 80	153 ± 83		
Placebo (*n* = 9)	163 ± 86	168 ± 84	136 ± 67		
Creatinine (mg/dL)				0.284	0.522
Pentaglobin (*n* = 10)	1.1 ± 0.7	1.0 ± 0.5	0.9 ± 0.4		
Placebo (*n* = 9)	1.9 ± 1.0	2.1 ± 1.6	1.8 ± 1.2		
Bilirubin (mg/dL)					–
Pentaglobin (*n* = 10)	0.7 [0.5–1.7]	0.8 [0.5–1.6]	0.9 [0.5–1.7]	0.356	
Placebo (*n* = 9)	1.0 [0.5–1.3]	0.9 [0.5–2.3]	0.7 [0.6–2.4]	0.515	
Glasgow Coma Scale					–
Pentaglobin (*n* = 10)	14 [13–15]	15 [13–15]	15 [11–15]	0.999	
Placebo (*n* = 9)	14 [10–14]	13 [9–14]	12 [10–14]	0.999	
SOFA score					–
Pentaglobin (*n* = 10)	9 [7–11]	9 [5–10]	7 [6–8]	0.229	
Placebo (*n* = 9)	10 [7–13]	12 [6–12]	10 [6–12]	0.544	
Propofol (mg/kg/h, *n*)					–
Pentaglobin (*n* = 10)	1.2 [0–2.4], 7	0 [0–2.6], 4	0 [0–2.6], 4	0.790	
Placebo (*n* = 9)	0.8 [0–1.6], 5	0 [0–1.1], 4	0 [0–1.6], 3	0.518	
Remifentanil (mcg/kg/min, *n*)					–
Pentaglobin (*n* = 10)	0.08 [0.04–0.09], 8	0.05 [0–0.08], 7	0.06 [0–0.10], 7	0.366	
Placebo (*n* = 9)	0.06 [0–0.10], 8	0.05 [0–0.08], 7	0.06 [0–0.10], 7	0.991	

Data are expressed as mean ± standard deviation or median [1st–3rd quartile], as appropriate

*MAP* mean arterial pressure, *HR* heart rate, *ScvO*_*2*_ central venous oxygen saturation, *SOFA* Sequential Organ Failure Assessment

^a^Two-way analysis of variance for repeated measures (testing the effect of time) or Friedman test, as appropriate

^b^Two-way analysis of variance for repeated measures (testing for the interaction between time and treatment), when applicable

* *p* < 0.05, **p < 0.01 versus baseline, Two-way analysis of variance for repeated measures with Sidack’s post hoc test or Friedman test with Dunn’s post hoc test for multiple comparisons, as appropriate

^#^*p* < 0.05 versus Pentaglobin group, Two-way analysis of variance for repeated measures with Sidack’s post hoc test or Mann–Whitney *U* test, as appropriate

**Table 4:** White blood cells, procalcitonin and cytokine levels

	Baseline	24 h	72 h	*p* (Friedman test)
White blood cell count (n/mmc)				
Pentaglobin (*n* = 10)	9070 [5560–18,660]	10,710 [6260–13,790]	10,100 [7388–11,720]	0.763
Placebo (*n* = 9)	12,000 [6145–23,300]	12,560 [8185–25,090]	12,990 [7275–26,220]	0.569
Procalcitonin (ng/mL)				
Pentaglobin (*n* = 10)	14.4 [3.4–48.6]	14.6 [4.8, 28.8]	7.1 [3.2–14.9]*	0.026
Placebo (*n* = 9)	20.0 [4.5–95.1]	19.7 [4.6–79.9]	5.2 [2.4–33]**	< 0.001
Interleukin-1 BETA (pg/mL)				
Pentaglobin (*n* = 10)	5.3 [4–12.8]	4.5 [4–6.7]	4 [4–5.6]	0.057
Placebo (*n* = 9)	4 [4–5.6]	4 [4, 5]	4 [4–5.8]	0.376
Tumour necrosis factor alpha (pg/mL)				
Pentaglobin (*n* = 10)	32 [20–84]	18 [14–38]	16 [12–28]	0.078
Placebo (*n* = 9)	30 [24–77]	39 [25–51]^#^	19 [16–42]	0.010
Interleukin-6 (pg/mL)				
Pentaglobin (*n* = 10)	350 [104–1418]	166 [61–781]	151 [41–296]*	0.030
Placebo (*n* = 9)	212 [52–971]	98 [36–217]	69 [21–141]	0.154
Interleukin-8 (pg/mL)				
Pentaglobin (*n* = 10)	138 [52–1268]	74 [29–177]	75 [49–108]	0.262
Placebo (*n* = 9)	146 [66–302]	62 [32–79]	57 [45–115]	0.278
Interleukin-10 (pg/mL)				
Pentaglobin (*n* = 10)	30 [10–118]	9 [5–15]*	8 [6–13]**	0.003
Placebo (*n* = 9)	20 [8–71]	19 [6–29]	10 [6–13]	0.685

Data are expressed as median [1st–3rd quartile]

* *p* < 0.05, **p < 0.01 versus baseline, Friedman test with Dunn’s test for multiple comparisons

No correlation was found between changes (delta 72 h-baseline) in PVDs and changes in MAP (Pearson *r* = − 0.073, *p* = 0.765), norepinephrine dose (*r* = 0.325, *p* = 0.175), ScvO_2_ (*r* = 0.171, *p* = 0.483) and cytokine levels (Spearman rho for TNF-alpha = − 0.125, *p* = 0.610; Il-6 = − 0.040, *p* = 0.870; Il-10 = − 0.146, *p* = 0.552).

No unintended effects were reported for any of the two study groups.

## Discussion

Microcirculatory dysfunction plays a key role in the pathogenesis of sepsis [19–22]. Persistent microcirculatory alterations during septic shock are associated with organ failure and death [23, 24]. In this single-centre, randomized, double-blind, placebo-controlled phase II trial, we showed that a 72-h infusion of IgM-enriched immunoglobulins (Pentaglobin) as an adjunctive therapy during sepsis may be associated with an increase in the sublingual microvascular density and blood flow quality. These changes did not correlate with variations in macro-hemodynamic parameters or cytokine levels. Although exploratory, these data would support a potential role of Pentaglobin therapy in favouring microvascular recruitment and tissue perfusion during sepsis.

A number of clinical studies suggested a beneficial effect of IgM-enriched immunoglobulins in sepsis; however, the quality of the available evidence remains low [7]. The use of immunoglobulins was introduced with the rationale of modulating the inflammatory reaction and supporting the immune system in the fight against pathogens [25]. In septic pigs, the infusion of Pentaglobin was able to shift the inflammatory response towards an anti-inflammatory profile [26]. In experimental sepsis models, Pentaglobin normalized capillary perfusion at 24 h by reducing venular leukocyte adhesion [10] and alleviated the histopathological injury in the lungs and small intestine [27, 28]. In a rat model of pneumonia, IgM-enriched immunoglobulins enhanced the anti-inflammatory response by increasing blood IL-10 levels and reducing TNF-alpha in bronchoalveolar lavage fluid [29]. In this study, we failed to detect a clear impact of Pentaglobin on the cytokine profile. The heterogeneity of sepsis syndrome likely influenced the variation in cytokine levels. In addition, we could have missed changes in cytokines occurring earlier than the first 24 h of treatment. Our study was not powered to detect changes in cytokine levels, which are extremely variable during sepsis.

Although it cannot be excluded that macro-hemodynamic changes unrelated to immunoglobulin therapy were responsible for the observed variations in the microcirculation, we could not find any correlation between changes in microvascular perfusion and variations of macro-hemodynamic parameters. While the infusion of Pentaglobin was able to improve the sublingual microcirculation without inducing any significant change in MAP or vasopressor dose, in the placebo group the PVD and MFI were reduced at 72 h despite a decrease in norepinephrine requirements. This loss of coherence between the macro- and the microvascular responses has been described during sepsis and shock states [30] and emphasizes that targeting systemic hemodynamic parameters may not be sufficient to ensure an optimization of tissue perfusion.

Pentaglobin had no consistent impact on tissue oxygenation and microvascular reactivity as assessed by NIRS. Thenar NIRS with a VOT enables to investigate alterations in tissue oxygen delivery and consumption, and to test the microvascular reserve capacity following a short period of ischemia. Reduced StO_2_ and slower StO_2_ downslopes and upslopes are generally found during sepsis and are associated with worse outcome [31]. Nonetheless, NIRS shows alterations in peripheral (skeletal muscle) oxygenation and may not be sensitive enough to detect a hypoperfusion in inner organs, whereas the capillary perfusion of the sublingual mucosa is generally explored as a window to the splanchnic microcirculation [32].

Our study has several limitations. First, the small sample size carries a high risk of type-I error. The study may be underpowered to detect differences in some parameters (including cytokine levels). Moreover, the two groups may be unbalanced for some baseline characteristics (e.g. norepinephrine dose and lactate levels). Therefore, our results should be considered as exploratory and need confirmation by future studies. No differences were observed in the SOFA score between the groups: unfortunately, however, our study was not powered to detect differences in mortality or other major outcomes (organ failures, shock reversal, ICU length of stay). Second, the comparison of absolute PVD values at 72 h (as per pre-planned statistical analysis) did not reach statistical significance. However, we believe that this is (at least partly) due to a between-group variability at baseline. In order to control for this confounder, we included a comparison of delta values (adjusted for baseline) that confirmed the different trend observed in the two groups. Third, most of the enrolled patients were already hemodynamically stable and less than 30% were in shock based on the Sepsis-3 definitions [12]. Consistently, we did not find severe microvascular alterations at baseline: an MFI < 2.6 [16] was observed only in 5 patients out of 19 and the PPV was < 90% only in 4 cases. As the infusion of Pentaglobin could produce a bigger effect in patients with more severe microcirculatory dysfunction, the presence of an altered microcirculation should be among the inclusion criteria in future studies. Fourth, we did not measure baseline immunoglobulin levels. The infusion of IgM-enriched immunoglobulins could have been more effective in patients with more severe hypo-IgG or hypo-IgM, who may represent the best target for this immunomodulatory therapy [33]. Lastly, data of cardiac output were not evaluated as available only for a small number of patients.

## Conclusions

In this single-centre, randomized, double-blind, placebo-controlled, phase II trial, a 72-h infusion of IgM-enriched immunoglobulins (Pentaglobin) in patients with sepsis or septic shock was associated with an increase in sublingual microvascular perfusion. Given the small sample size, these results must be seen as exploratory and need to be confirmed by other studies.

## Supplementary information

**Additional file 1.** Comparison of changes in sublingual microcirculation and NIRS-derived parameters.

## Data Availability

The datasets used and/or analysed during the current study are available from the corresponding author on reasonable request.

## Abbreviations

IgM: immunoglobulins M
PVD: perfused vessel density
MAP: mean arterial pressure
HR: heart rate
Hb: haemoglobin
WBC: white blood cells
IL: interleukin
TNF-α: tumour necrosis factor alpha
SAPS: Simplified Acute Physiology Score
APACHE: Acute Physiology and Chronic Health Evaluation
SOFA: Sequential Organ Failure Assessment
IDF: Incident Dark Field
TVD: total vessel density
PPV: percentage of perfused vessels
MFI: microvascular flow index
FHI: flow heterogeneity index
NIRS: near-infrared spectroscopy
StO_2_: tissue O_2_ saturation
THI: tissue haemoglobin index
VOT: vascular occlusion test
AUC: area under the curve
ANCOVA: analysis of covariance
ScvO_2_: central venous O_2_ saturation
