# Supplementary material for: IgM-enriched immunoglobulins (Pentaglobin) may improve the microcirculation in sepsis: a pilot randomized trial
Source: Ann Intensive Care. 2019 Dec 3;9:135. doi: 10.1186/s13613-019-0609-5 (PMC6890901; doi:10.1186/s13613-019-0609-5)
Supplement: Supplementary file 1 — Additional file 1. Comparison of changes in sublingual microcirculation and NIRS-derived parameters. [file 13613_2019_609_MOESM1_ESM.docx]

**Additional File 1– Comparison of changes in sublingual microcirculation and NIRS-derived parameters.**

| **Microcirculatory Parameters** | **Baseline** | **24 h (change from baseline)** | **72 h (change from baseline)** | **Adjusted p (time*group, RM ANCOVA)^a^** |
| --- | --- | --- | --- | --- |
| PVDs (mm/mm^2^) |  |  |  | 0.003 |
| Pentaglobin (n=10) | 21.7 (4.7) | +1.7 (3.4) | +3.8 (3.8)** |  |
| Placebo (n=9) | 25.0 (5.8) | -1.2 (4.0) | -4.2 (4.7) |  |
| MFIs (AU) |  |  |  | 0.035 |
| Pentaglobin (n=10) | 2.6 (0.3) | +0.3 (0.2) | +0.2 (0.2)** |  |
| Placebo (n=9) | 2.8 (0.2) | 0 (0.3) | -0.2 (0.2) |  |
| PPVs (%) |  |  |  | 0.346 |
| Pentaglobin (n=10) | 94 (5)^#^ | +4.8 (4.5) | +4.4 (5.8) |  |
| Placebo (n=9) | 97 (2) | +0.2 (2.7) | -1.5 (3.2) |  |
| TVDs (mm/mm^2^) |  |  |  | 0.093 |
| Pentaglobin (n=10) | 23.3 (4.9) | +0.6 (3.5) | +1.9 (4.8) |  |
| Placebo (n=9) | 25.6 (5.9) | -1.2 (3.8) | -3.2 (5.1) |  |
| De Backer score (n/mm) |  |  |  | 0.221 |
| Pentaglobin (n=10) | 13.1 (2.2) | +0.2 (1.8) | +1.0 (2.2) |  |
| Placebo (n=9) | 13.9 (2.8) | -0.7 (2.1) | -1.1 (2.2) |  |
| FHI (AU) |  |  |  | 0.680 |
| Pentaglobin (n=10) | 0.3 (0.2) | -0.3 (0.2) | -0.2 (0.2) |  |
| Placebo (n=9) | 0.2 (0.1) | 0 (0.2) | 0 (0.2) |  |
| StO_2_ (%) |  |  |  | 0.473 |
| Pentaglobin (n=10) | 80 (6) | +3.2 (6.5) | +2.0 (7.6) |  |
| Placebo (n=9) | 79 (10) | -0.2 (5.3) | +1.5 (8.1) |  |
| StO_2_ Downslope (%/min) |  |  |  | 0.523 |
| Pentaglobin (n=10) | -10.0 (6.4) | -0.4 (5.0) | -11.8 (4.9) |  |
| Placebo (n=9) | -9.9 (5.4) | -0.5 (2.2) | -9.8 (4.1) |  |
| StO_2_ Upslope (%/min) |  |  |  | 0.595 |
| Pentaglobin (n=10) | 157 (37) | +66 (73) | +49 (78) |  |
| Placebo (n=9) | 120 (57) | +42 (37) | +27 (57) |  |
| Area of hyperemia (%*min) |  |  |  | 0.188 |
| Ig-GAM (n=10) | 16.7 (10.5) | -4.0 (8.3) | -6.4 (12.1) |  |
| Placebo (n=9) | 15.3 (10.3) | -1.4 (4.8) | -8.2 (9.3) |  |
| THI (AU) |  |  |  | 0.858 |
| Pentaglobin (n=10) | 11.9 (2.9) | -1.5 (3.3) | -1.8 (3.9) |  |
| Placebo (n=9) | 10.5 (3.7) | -1.1 (3.7) | -0.7 (4.3) |  |

Data are expressed as mean (standard deviation).

^a^ Adjusted for baseline value of the outcome of interest. #p<0.05 versus placebo, Student t test; **p<0.01 versus placebo, Repeated measure ANCOVA with Bonferroni post hoc test for between-group comparisons

*NIRS* Near Infrared Spectroscopy, *RM ANCOVA* analysis of covariance for repeated measures, *TVD* total vessel density, *PVD* perfused vessel density, *PPV* percentage of perfused vessels, *MFI* microvascular flow index, *FHI* flow heterogeneity index, *StO2* tissue oxygen saturation, *THI* tissue haemoglobin index, *AU* arbitrary units
